# Mapping malaria hotspots through spatial and spatio-temporal analysis in Sierra Leone, 2021–2024

**DOI:** 10.5588/pha.25.0048

**Published:** 2026-05-18

**Authors:** A.M. Falama, O. Omoniwa, M.S. Kanu, W.K. Lahai, A.R.Y. Kamara, G. Ameh, I.F. Kamara, B.D. Fofanah, S. Kenneh, M.N. Kamara, M.A. Sesay, N. Sesay, F. Kanu, A.A. Alwani, P. Thekkur, R. Zachariah, M.S. Kanu, S. Lakoh

**Affiliations:** 1 National Malaria Control Programme, Ministry of Health, Freetown, Sierra Leone;; 2 World Health Organization Office, Freetown, Sierra Leone;; 3 Ministry of Health, Government of Sierra Leone, Freetown, Sierra Leone;; 4 Centre for Operational Research, International Union Against Tuberculosis and Lung Disease (The Union), Paris, France;; 5 The Union South East Asia Office, New Delhi, India;; 6 UNICEF, UNDP, World Bank, WHO Special Programme for Research and Training in Tropical Diseases (TDR), Geneva, Switzerland;; 7 Informatics Consultancy Firm, Freetown, Sierra Leone.

**Keywords:** malaria, incidence, geospatial analysis, space–time cluster

## Abstract

**SETTING:**

Sierra Leone, using data from the District Health Information System 2 (DHIS2) database.

**OBJECTIVE:**

This study examined the spatial and spatio-temporal distribution of malaria incidence, and the relationship between malaria incidence and rainfall in surrounding areas in Sierra Leone from 2021 to 2024.

**METHOD:**

A cross-sectional geospatial study was conducted using malaria case data, mean rainfall data, population estimates, and chiefdom-level geographic coordinates. Spatial clustering was evaluated using Moran’s I, significant district-level clusters were identified through space–time Poisson models (α = 0.05; 999 permutations), and the relationship between malaria incidence and rainfall in surrounding areas was assessed using bivariate Moran’s I, implemented in Python.

**RESULTS:**

Between 2021 and 2024, 7.4 million malaria cases were reported. Chiefdom-level incidence ranged from 21.2 to >750 per 1,000 population. Significant spatial clustering was observed (Moran’s I > 0; *P* < 0.01). Persistent high–high clusters (*P* < 0.05) and low–low clusters (*P* < 0.05) were identified across the country. Space–time analysis identified both high-risk (relative risk [RR] = 1.2–1.9; *P* < 0.01) and low-risk districts (RR = 0.5–0.9; *P* < 0.01). There was no significant association between malaria incidence and rainfall in surrounding areas at the national level.

**CONCLUSION:**

Malaria transmission remains spatially and temporally heterogeneous, with persistent hotspots that require tailored subnational interventions to accelerate progress towards elimination.

Malaria remains a leading cause of morbidity and mortality in many developing countries, particularly in sub-Saharan Africa, where it poses a significant public health challenge.^1^ Despite a decrease in the malaria burden between 2005 and 2015, there has been a recent resurgence of cases. In 2024, an estimated 282 million cases occurred globally, the highest number in the past 25 years. The annual number of deaths has plateaued over recent years and is estimated at around 600,000 per year. The African region carries a disproportionately high share of the global malaria burden, accounting for 95% of cases and deaths.^1^

Sierra Leone, a country on the western coast of Africa, has only 0.1% of the world’s population but contributes almost 1% of global malaria cases.^1^ In 2023, more than 2 million cases were reported, and an additional 0.5 million estimated.^2^ Malaria is the leading cause of mortality in the country, accounting for 2,637 deaths in 2023.^2,3^ To combat the disease, the country’s National Malaria Control Program (NMCP) is implementing the National Malaria Elimination Strategic Plan for 2021–2025.^4^ The strategy aligns with the WHO Global Technical Strategy (GTS), which calls for reductions in malaria case incidence and mortality rates of at least 75% by 2025.^5^ Despite a decrease in cases from 2.75 million in 2015 to 2.4 million in 2024,^1,2^ Sierra Leone is not on track to meet the 2025 targets; and if the current trends continue, the 2030 GTS targets of reduction by 90% are also unlikely to be achieved, unless efforts are accelerated. Transmission of malaria depends on several factors, including climate, geographical terrain, urbanisation, access to health care, and the coverage of malaria interventions, resulting in spatial and temporal heterogeneity in its burden.^6,7^ This leads to the emergence of geographical hotspots that change periodically. Research has shown that interventions targeted at high-burden areas lead to reductions in morbidity and mortality and are more cost-effective than mass strategies for malaria control.^8,9^ Identifying and targeting prevention and control efforts in these hotspots could accelerate progress towards elimination.

In Sierra Leone, malaria control activities are decentralised to the district and chiefdom levels; therefore, identifying hotspots at these levels would provide actionable insights for focusing intervention efforts. Geospatial analysis, utilising Geographic Information Systems (GISs) and remote sensing technologies to visualise, analyse, and interpret data related to the geographical aspects of health issues, provides an opportunity to map malaria cases and identify transmission hotspots. Because such information supports subnational targeting of malaria interventions, this study examined whether significant spatial and space–time clusters of malaria occurred in Sierra Leone from 2021 to 2024, and aimed to identify these clusters. Additionally, it assessed the relationship between malaria incidence and rainfall in surrounding areas. Rainfall is a key environmental determinant of malaria transmission, influencing vector breeding and survival, and its effects may extend across neighbouring locations rather than being confined to administrative boundaries. Examining the spatial relationship between malaria incidence and rainfall helps clarify whether persistent malaria clusters are linked to rainfall or other influencers.

## METHODS

We employed a cross-sectional, geospatial analysis utilising data sourced from the District Health Information System 2 (DHIS2) and projections of the population census of 2015.^10^

### General study setting

Sierra Leone covers a total area of 71,740 km^2^, with a coastline of 402 km and a projected population of 8.9 million, distributed across five regions, 16 districts, and 190 chiefdoms.^10^ The country’s terrain is varied, featuring swamps, rain forests, and one of West Africa’s highest mountains, the 2,200 m Bintumani. The coastline of the country has several mangrove swamps, which provide breeding sites for the *Anopheles melas* mosquito, one of the major vectors of malaria in these specific habitats. The country experiences a typical tropical climate, with temperatures ranging from 21°C to 32°C, and an average daily temperature of 25°C. There are two main seasons: the wet season (May to October) and the dry season (November to April), with heavy rainfall occurring in July and August. The average rainfall is about 320 mm annually, and relative humidity is high, ranging from 60% to 90%.^11^ Analysis of time trends using malaria case data from Sierra Leone shows that the highest number of cases occur in June and July, coinciding with the onset of the rainy season, and the lowest number of cases occur in February, when mosquito populations decline.^12^

### Specific study setting

The health system in Sierra Leone is decentralised, with District Health Management Teams implementing health programmes at the district level. Malaria diagnosis and treatment are provided at all levels of care. Suspected cases (febrile patients) are tested using malaria rapid diagnostic tests or microscopy, with positive results confirming infection. Uncomplicated malaria is managed on an outpatient basis at hospitals, at peripheral health units, and in the community by the community health workers (CHWs), whereas severe malaria cases are admitted and treated at secondary and tertiary facilities. CHWs, public facilities, and selected private facilities collect data on malaria cases and record it in outpatient and inpatient registers. They report aggregated data monthly and enter it into the DHIS2 platform. The NMCP can access, monitor, and review the information through this platform. In 2023, 80% of estimated cases were notified.^2^

### Data variables, extraction, and analysis

Malaria case data: The number of malaria cases reported annually from 2021 to 2024 for each district and chiefdom was extracted from the DHIS2 platform.

Population data: Chiefdom- and district-level population estimates for the respective years were extracted from the DHIS2. These estimates are based on the 2015 national population census,^10^ using a projected annual growth rate of 2.5%.

Rainfall data: Chiefdom average monthly rainfall data were extracted from the Climate Hazards Group InfraRed Precipitation with Station (CHIRPS) data base.

Geographic location data: Chiefdom shapefiles to conduct the GIS analysis, along with geo-coordinates, were obtained from DHIS2.

Malaria case, rainfall, population, and coordinate datasets were exported into Microsoft open format spread sheet (xlsx format). All data were fully de-identified, and the electronic files were stored on a password-protected computer accessible only to the study team. All datasets were cleaned, harmonised, and checked for completeness prior to analysis. For each chiefdom, the annual malaria crude incidence proportion (CIP) per 1,000 population for 2021–2024 was calculated by dividing the yearly number of reported cases by the chiefdom population and multiplying by 1,000. Chiefdom-level incidence was then mapped using WHO-aligned incidence classes to allow consistent comparison across years.^13^ Spatial autocorrelation was assessed for each year at the district level using Moran’s I with permutation-based significance testing. Local Moran’s I was then used to identify statistically significant high–high clusters, low–low clusters, and outlier areas, with non-significant locations masked. Space–time patterns were evaluated by aggregating chiefdom-level results to districts. Departures from expected cases were tested each year using a Poisson model to generate relative risks (RRs), directional *P* values, and cluster labels indicating high risk (RR > 1), low risk (RR < 1), or non-significant risk. District-level sequences from 2021 to 2024 were reviewed to identify persistent, emerging, or intermittent transmission patterns. The relationship between malaria incidence and rainfall in surrounding areas was assessed using bivariate Moran’s I, with spatial lags calculated based on queen contiguity neighbours. Significance was evaluated using permutation tests, and persistent high-incidence–high-rainfall chiefdoms were identified across years. All spatial and statistical analyses were conducted in Python using standard geographic and analytical libraries.

### Ethical statement

Ethical approval was obtained from the Sierra Leone Ethics and Scientific Review Committee of the Ministry of Health (SLESRC No. 012/02/2025; approved 26 February 2025). Permission to use the data was obtained from the Chief Medical Officer of the Ministry of Health.

## RESULTS

### Spatial distribution of malaria incidence at chiefdom level

**FIGURE 1.**
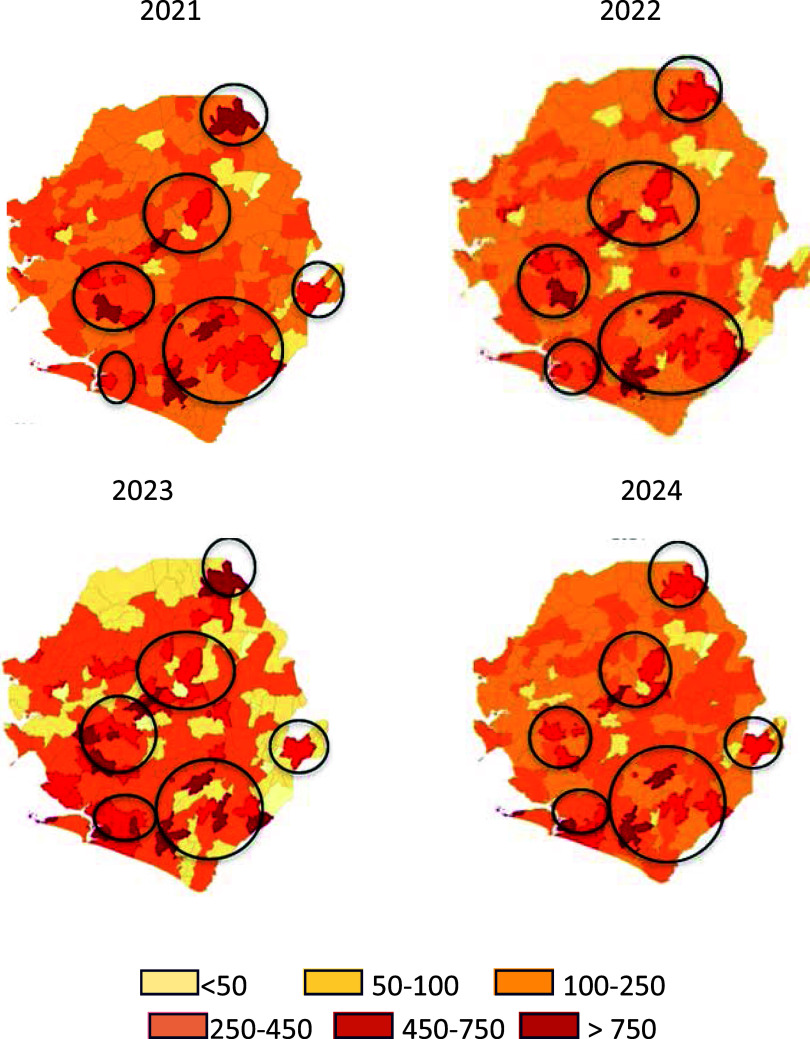
Chiefdom-level crude malaria incidence in Sierra Leone, 2021–2024.

Between 2021 and 2024, a total of 7,387,409 malaria cases were report across all the chiefdoms in Sierra Leone. The highest number of cases occurred in 2023 (2,130,356), followed by 2021 (1,908,438) and 2022 (1,739,856), while 2024 reported the lowest number of cases (1,608,759). There was a marked spatial variation in the chiefdom-level CIP between 2021 and 2024. Most chiefdoms consistently fell within the 100–450 cases per 1,000 population range, reflecting widespread moderate-to-high transmission (Figure 1). Very low–incidence areas (<50 per 1,000) were uncommon, whereas a few chiefdoms in Bo, Moyamba, Pujehun, and Tonkolili districts exhibited extremely high incidence (>750 per 1,000) during the 4-year period. High-incidence chiefdoms (>250 per 1,000 population) persisted across the 4 years, especially in the southern part of the country, indicating areas of stable transmission (Figure 1).

### Spatial clustering of malaria cases

**FIGURE 2.**
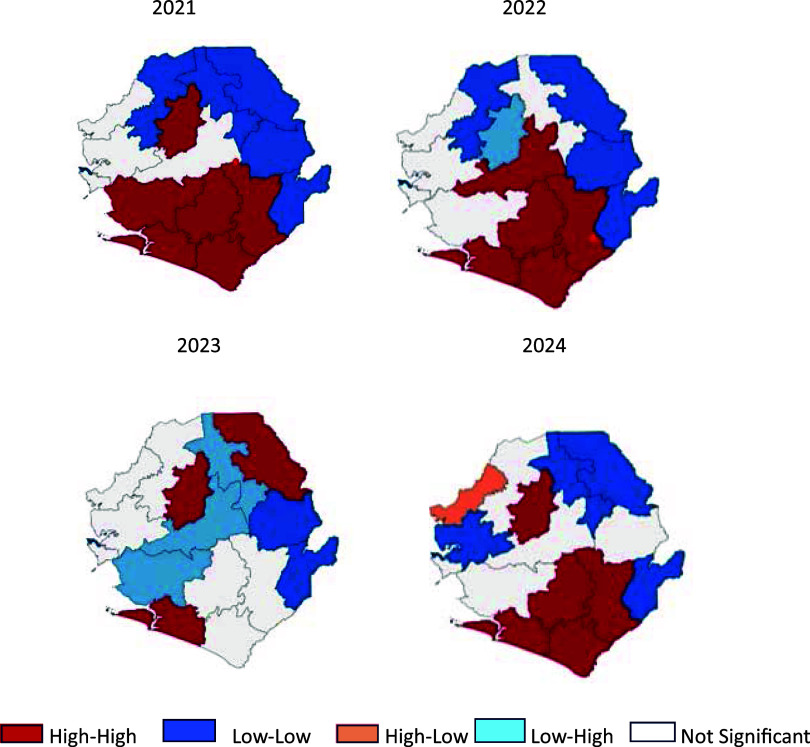
District-level Local Moran’s I clustering of malaria incidence in Sierra Leone, 2021–2024.

Local Moran’s I cluster categories at the district level, by year, showed high–high (hotspots), low–low (coldspots), high–low, and low–high spatial patterns (Figure 2). The results of spatial autocorrelation from 2021 to 2024 revealed that Bo, Bonthe, Moyamba, Tonkolili, and parts of Kenema frequently appeared as high–high clusters, indicating districts with high malaria incidence surrounded by similarly high-incidence neighbours. These clusters represent zones of sustained malaria transmission. In contrast, Bombali, Karene, Falaba, Koinadugu, Kono, and Kailahun commonly appeared as low–low clusters, indicating comparatively lower incidence within spatially cohesive areas. High–low and low–high outlier patterns appeared intermittently, signalling sharp local deviations from neighbouring districts (Table 1; Figure 2).

**TABLE 1.** Spatial clustering of annual crude malaria incidence across districts in Sierra Leone, 2021–2024 (Local Moran’s I).

District	Neighbours	2021		2022		2023		2024	
		Lisa	*P* value	Lisa	*P* value	Lisa	*P* value	Lisa	*P* value
Bo	Bon, Ken, Moy, Puj, Ton	H-H	0.007*	H-H	0.004*	H-H	0.006*	H-H	0.005*
Bombali	Kar, Koin, PL Ton	L-L	0.164	L-L	0.297	L-L	0.496	L-L	0.140
Bonthe	Bo, Moy, Puj	H-H	0.040*	H-H	0.050*	H-H	0.090	H-H	0.045*
Falaba	Koin, Kon	L-L	0.097	L-L	0.099	L-L	0.331	L-L	0.353
Kailahun	Ken, Kon	L-L	0.469	L-L	0.406	L-L	0.234	L-H	0.490
Kambia	Kar, PL	H-L	0.180	H-L	0.264	H-L	0.224	H-L	0.028*
Karene	Bom, Kam, Koin, PL	L-L	0.175	L-L	0.232	L-L	0.382	L-L	0.066
Kenema	Bo, Kai, Kon, Puj, Ton	H-L	0.452	H-L	0.409	L-L	0.434	L-H	0.204
Koinadugu	Bom, Fal, Kar, Kon, Ton	L-L	0.052	L-L	0.064	H-L	0.198	L-L	0.140
Kono	Fal, Kai, Ken, Koin, Ton	L-L	0.135	L-L	0.130	L-L	0.186	H-L	0.125
Moyamba	Bo, Bon, PL, Ton, WAR	H-H	0.017*	H-H	0.020*	H-H	0.018*	L-H	0.039*
Port Loko	Bom, Kam, Kar, Moy Ton, WAR	L-H	0.433	L-H	0.432	L-H	0.303	L-L	0.345
Pujehun	Bo, Bon, Ken	H-H	0.001*	H-H	0.003*	L-H	0.037*	H-H	0.024*
Tonkolili	Bo, Bom, Ken, Koin, Kon, Moy, PL	H-H	0.353	H-L	0.426	H-L	0.459	H-L	0.244
WAR	Moy, PL, WAU	H-L	0.472	H-L	0.407	H-L	0.366	H-L	0.182
WAU	WAR	L-H	0.338	L-H	0.405	L-H	0.336	L-H	0.338

LISA = Local Indicators of Spatial Association; Bom = Bombali; Bon = Bonthe; Fal = Falaba; Kam = Kambia; Kai = Kailahun; Kar = Karene; Ken = Kenema; Koin = Koinadugu; Kon = Kono; Moy = Moyamba; PL = Port Loko; Puj = Pujehun; Ton = Tonkolili; WAR = Western Area Rural; WAU = Western Area Urban; H-H = High–High; L-L = Low–Low; H-L = High–Low; L-H = Low–High.

*Significant *P* value.

### Temporal clustering of malaria cases by district

Application of population-adjusted Poisson models identified district-year combinations with significantly high or low malaria risk relative to expected counts. Several districts demonstrated persistent excess risk (RR > 1) across all 4 years, including Bo, Bonthe, Tonkolili, Kambia, Kenema, and Western Rural, with RRs ranging from 1.2 to 1.9 (Table 2). These districts consistently recorded substantially more cases than expected, indicating stable and intense transmission. On the other hand, Bombali, Karene, Falaba, Kono, and Kailahun exhibited persistently suppressed malaria risk (RR < 1), with values ranging from 0.5 to 0.9, suggesting stable coldspots over time. A few districts transitioned between high and low risk across the 4 years, most notably, Pujehun (high in 2022 and 2024; low in 2021 and 2023) and Koinadugu (high in 2023 only).

**TABLE 2.** Space–time Poisson analysis of district-level malaria clustering in Sierra Leone, 2021–2024.

District	2021					2022					2023					2024				
	Obs	Exp	RR	Cluster	*P* value	Obs	Exp	RR	Cluster	*P* value	Obs	Exp	RR	Cluster	*P* value	Obs	Exp	RR	Cluster	*P* value
Bo	169,645	107,035	1.58	High	<0.001	138,237	96,864	1.43	High	<0.001	155,522	124,835	1.25	High	<0.001	133,712	100,868	1.33	High	<0.001
Bombali	94,774	118,043	0.80	Low	<0.001	81,241	106,826	0.76	Low	<0.001	120,431	137,673	0.87	Low	<0.001	87,173	111,242	0.78	Low	<0.001
Bonthe	87,807	57,745	1.52	High	<0.001	86,685	52,258	1.66	High	<0.001	126,142	67,348	1.87	High	<0.001	105,180	54,418	1.93	High	<0.001
Falaba	87,232	114,253	0.76	Low	<0.001	75,451	103,396	0.73	Low	<0.001	93,480	133,254	0.70	Low	<0.001	64,048	107,671	0.59	Low	<0.001
Kailahun	81,680	151,824	0.54	Low	<0.001	67,798	137,397	0.49	Low	<0.001	105,388	177,073	0.60	Low	<0.001	94,498	143,077	0.66	Low	<0.001
Kambia	116,126	99,359	1.17	High	<0.001	108,411	89,917	1.21	High	<0.001	141,767	115,882	1.22	High	<0.001	100,475	93,635	1.07	High	<0.001
Karene	66,246	82,124	0.81	Low	<0.001	66,905	74,320	0.90	Low	<0.001	86,054	95,782	0.90	Low	<0.001	58,382	77,393	0.75	Low	<0.001
Kenema	226,643	175,406	1.29	High	<0.001	203,964	158,738	1.28	High	<0.001	205,632	204,576	1.01	High	0.010	167,571	165,301	1.01	High	<0.001
Koinadugu	49,930	58,676	0.85	Low	<0.001	46,726	53,100	0.88	Low	<0.001	72,454	68,434	1.06	High	<0.001	46,668	55,296	0.84	Low	<0.001
Kono	102,954	144,964	0.71	Low	<0.001	92,060	131,189	0.70	Low	<0.001	140,587	169,072	0.83	Low	<0.001	146,005	136,612	1.07	High	<0.001
Moyamba	104,056	87,191	1.19	High	<0.001	85,901	78,906	1.09	High	<0.001	121,164	101,691	1.19	High	<0.001	75,482	82,168	0.92	Low	<0.001
Port Loko	127,524	152,679	0.84	Low	<0.001	117,235	138,170	0.85	Low	<0.001	147,148	178,069	0.83	Low	<0.001	92,431	143,883	0.64	Low	<0.001
Pujehun	83,327	89,149	0.93	Low	<0.001	84,840	80,677	1.05	High	<0.001	102,688	103,974	0.99	Low	<0.001	116,547	84,013	1.39	High	<0.001
Tonkolili	171,192	149,365	1.15	High	<0.001	165,281	135,171	1.22	High	<0.001	240,281	174,204	1.38	High	<0.001	185,417	140,760	1.32	High	<0.001
Western rural	146,866	127,774	1.15	High	<0.001	132,727	115,632	1.15	High	<0.001	162,997	149,022	1.09	High	<0.001	144,105	120,412	1.20	High	<0.001
Western urban	134,952	135,366	1.00	NS	0.131	121,604	122,503	0.99	Low	0.005	137,032	157,877	0.87	Low	<0.001	126,622	127,567	0.99	Low	0.004

Obs = observed number of cases; Exp = expected number of cases; RR = risk ratio; NS = not significant.

### Relationship between malaria incidence and rainfall in surrounding areas

**FIGURE 3.**
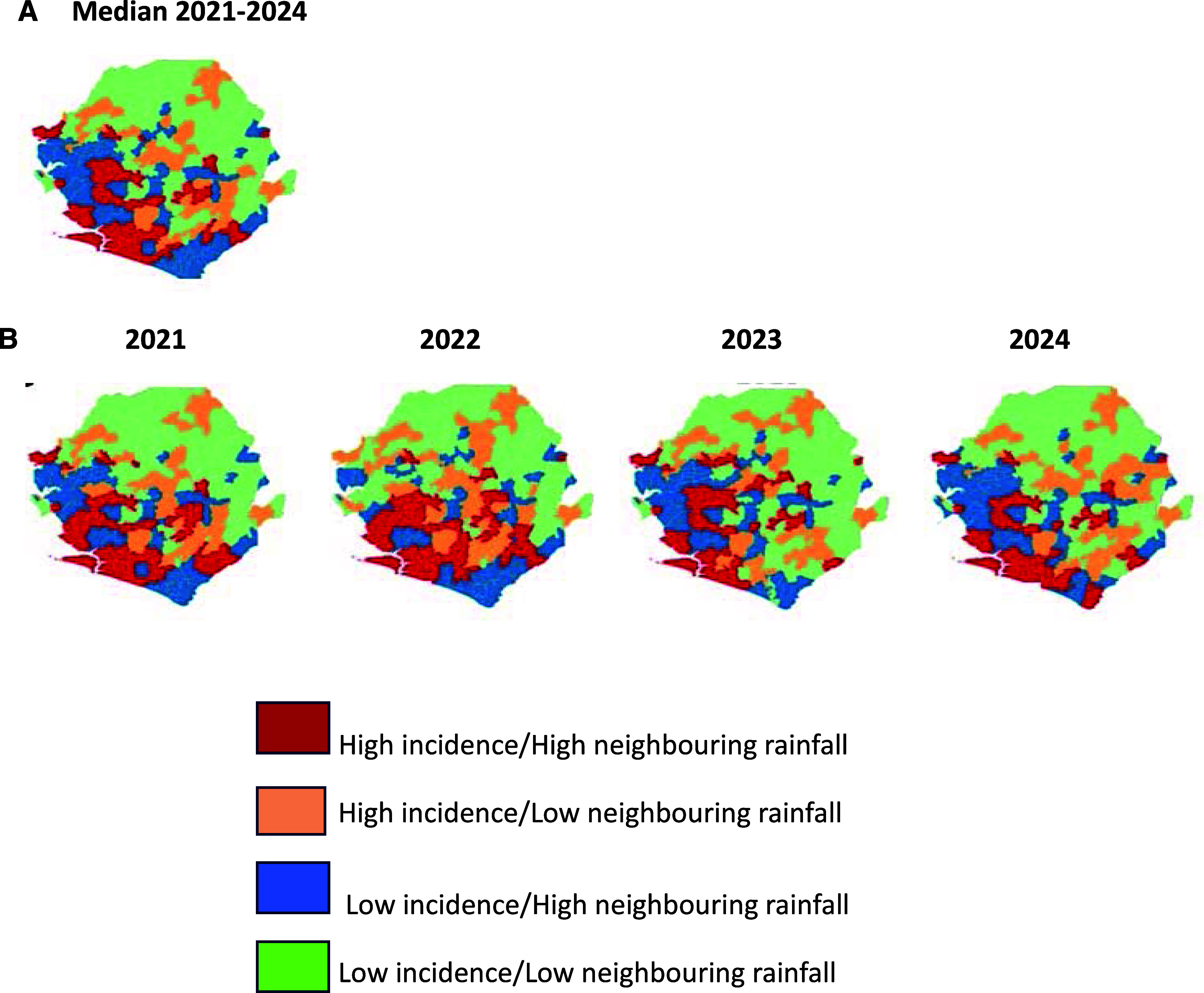
Chiefdom-level bivariate Moran’s I clusters of malaria incidence associated with neighbouring rainfall. **A)** Median in Sierra Leone, 2021–2024; **B)** annual incidence level and rainfall pattern, 2021–2024.

At the national level, the spatial association between malaria incidence and rainfall in surrounding areas was weak (global bivariate Moran’s I for 2021–2024 was 0.015) and not statistically significant (permutation *P* = 0.323), indicating that, overall, high-incidence areas were not consistently surrounded by high-rainfall areas. Annual analysis from 2021 to 2024 showed low, non-significant Moran’s I values of 0.01–0.04, suggesting that rainfall was not a dominant driver of malaria incidence in neighbouring chiefdoms across the 4 years. Local analyses revealed that several chiefdoms consistently exhibited a high–high pattern for 3–4 years between 2021 and 2024. These were concentrated in Moyamba, Bonthe, Kenema, Tonkolili, and Bo districts (4–6 chiefdoms per district). Other districts showed only one chiefdom with a persistent high–high pattern (Figure 3).

## DISCUSSION

This study employed spatial and spatio-temporal analyses to identify malaria hotspots across Sierra Leone from 2021 to 2024, providing empirical evidence of persistent transmission in specific geographic regions despite the nationwide scale-up of interventions. The findings revealed significant heterogeneity in malaria incidence, with the highest number of cases recorded in 2023 and a subsequent decline in 2024. This temporal fluctuation likely reflects a combination of epidemiological and programmatic factors. Reduced case reporting during 2021–2022 aligns with global disruptions in health care access and diagnostic services due to the COVID-19 pandemic.^14,15^ As routine service delivery recommenced in 2023, reported cases increased markedly, possibly indicating improved surveillance and the restoration of malaria testing capacity. The subsequent decrease observed in 2024 coincides with the national insecticide-treated net distribution campaign, consistent with prior studies demonstrating a reduction in malaria incidence following increased household ownership and utilisation of insecticide-treated nets.^16,17^

Spatial analysis highlighted stable, statistically significant clustering of malaria in Bo, Bonthe, Moyamba, Pujehun, Tonkolili, and parts of Kenema, indicating sustained transmission over time. These findings demonstrate that malaria transmission in Sierra Leone is not uniformly distributed but instead occurs in geographically concentrated foci that remain relatively stable over time. Similar spatial heterogeneity has been documented in several sub-Saharan African countries, including Zimbabwe, Ethiopia, and Kenya.^6,18,19^ In Sierra Leone, these hotspot regions share ecological and socio-environmental characteristics conducive to vector breeding, including high rainfall, dense vegetation, and the presence of mangrove or swamp habitats, mines, and farms that support *Anopheles melas* and *Anopheles gambiae* populations.^12^ Limited access to health care services and lower intervention coverage in remote chiefdoms may also contribute to ongoing transmission. Conversely, districts in the northern and eastern regions, such as Bombali, Karene, Falaba, Koinadugu, and Kailahun, exhibited consistent low–low clusters, indicating areas of comparatively reduced transmission. These spatial patterns align with previous observations that malaria risk in Sierra Leone diminishes with increasing altitude and improves with greater access to preventive and curative interventions.^20^

The persistence of high-risk zones across multiple years suggests entrenched transmission pockets that necessitate intensified, geographically targeted responses rather than uniform national strategies.^21^ From a programmatic standpoint, these results endorse a paradigm shift towards subnational stratification and targeted intervention deployment. Approaches such as indoor residual spraying, larval source management, enhanced surveillance in stable hotspots, and continuous preventive therapy for vulnerable populations should be prioritised in high-transmission chiefdoms.^8,9^ Meanwhile, maintaining core preventive interventions in low-transmission regions will be critical to prevent resurgence. These evidence-based, focal strategies align with the objectives of the upcoming National Malaria Elimination Strategic Plan 2026–2030 and the WHO GTS 2015–2030, both of which emphasise stratification and the efficient allocation of resources to accelerate progress towards malaria elimination.^5,22^

When planning malaria control strategies in hotspots, climatic factors in neighbouring areas should be considered, given their association with spatial spillover of malaria, potentially through human and vector mobility.^23^ Although no significant association was found between malaria incidence and surrounding rainfall at the national level in this study, high-incidence chiefdoms with both low- and high-rainfall neighbours were identified. From a control perspective, these findings suggest that interventions may benefit from being tailored to local environmental contexts: in chiefdoms with high malaria incidence and high surrounding rainfall, measures that reduce human–vector contact may be especially important rather than prioritising larval source management, as vectors may originate from surrounding areas.

While this analysis benefits from 4 years of nationally representative data and illustrates the power of routine surveillance for spatial decision-making, some limitations warrant acknowledgement. Population estimates relied on the 2015 census projections, which may not fully capture recent demographic shifts. Routine data may also under-report cases from private facilities and community-based sources, and the study did not differentiate between imported and locally acquired infections. Additionally, the analysis identified hotspots but did not explore the underlying determinants of clustering in detail, such as behavioural, intervention-specific, or additional environmental factors. Future research using advanced geospatial technologies should incorporate climate, vector, and intervention coverage data to elucidate causal drivers of hotspot persistence and to inform targeted malaria control strategies.

## CONCLUSION

This study demonstrates that malaria in Sierra Leone remains spatially clustered, with persistent high-risk areas despite ongoing interventions. Adopting dynamic subnational targeting and integrating spatial intelligence into programme planning will be crucial for achieving the country’s goal of malaria elimination.
